# Long-Chain Acylcarnitines Regulate the hERG Channel

**DOI:** 10.1371/journal.pone.0041686

**Published:** 2012-07-25

**Authors:** Fabio Ferro, Aude Ouillé, Truong-An Tran, Pierre Fontanaud, Patrick Bois, Dominique Babuty, François Labarthe, Jean-Yves Le Guennec

**Affiliations:** 1 INSERM U921, Université François-Rabelais, Tours, France; 2 INSERM U1046, Université Montpellier-1, Université Montpellier-2, Montpellier, France; 3 IGF-CNRS INSERM 661, Université Montpellier-1, Université Montpellier-2, Montpellier, France; 4 IPBC, UMR 6187, CNRS, Université de Poitiers, Poitiers, France; 5 CHRU Tours, Hôpital Trousseau, Service de cardiologie B, Tours, France; Indiana University School of Medicine, United States of America

## Abstract

**Background and purpose:**

In some pathological conditions carnitine concentration is high while in othersitis low.In bothcases,cardiac arrhythmiascan occur and lead to sudden cardiac death. It has been proposed that in ischaemia, acylcarnitine (acyl-CAR), but not carnitine, is involved in arrhythmiasthrough modulation of ionic currents. We studied the effects of acyl-CARs on hERG, K_IR_2.1 and K_v_7.1/minKchannels (channels responsible for I_KR_, I_K1_ and I_KS_ respectively).

**Experimental approach:**

HEK293 cells stably expressing hERG, K_IR_2.1 or Kv7.1/minK were studied using the patch clamp technique. Free carnitine (CAR) and acyl-CAR derivatives from medium- (C8 and C10) and long-chain (C16 and C18∶1) fatty acids were applied intra- and extracellularly at different concentrations. Forstudies onhERG, C16 and C18∶1 free fatty acid were also used.

**Key results:**

Extracellular long-chain (LCAC), but not medium-chain, acyl-CAR,induced an increase of I_hERG_ amplitude associated with a dose-dependent speeding of deactivation kinetics. They had no effect on K_IR_2.1 or Kv7.1/minK currents.Computer simulations of these effects wereconsistent with changes in action potential profile.

**Conclusions and applications:**

Extracellular LCAC tonically regulates I_hERG_ amplitude and kineticsunder physiological conditions. This modulation maycontribute tothe changes in action potential duration thatprecede cardiac arrhythmias in ischaemia, diabetes and primary systemic carnitine deficiency.

## Introduction

Fatty acids are the primary substrate used by the heart to generate ATP [1]. The initial steps involved in ATP production via β-oxidation include the formation of essential intermediates that facilitate the transport of fatty acids into the mitochondrial matrix where the β-oxidation enzymes are located. More specifically, after their entry into the cell, fatty acids are activated in the cytosol via the formation of acyl-CoA derivatives, which are either incorporated into intracellular lipid pools or enter mitochondria for β-oxidation. Carnitine (CAR) is the essential co-factor needed for mitochondrial fatty acid oxidation. It operates by shuttling long-chain fatty acids as acylcarnitinesesters (acyl-CAR) from the cytoplasm across the inner mitochondrial membrane into the mitochondrial matrix. After their entry into the mitochondria, long-chain acyl-CAR(LCAC) are converted back to long-chain acyl-CoAsesters, which are then β-oxidized into acyl-CoA of progressively shorter chain lengths (i.e. long-, medium-, short-chain acyl-CoA, and ultimately acetyl-CoA). Free carnitineis then recycled [2]. In contrast to acyl-CoA, acyl-CARs diffuse easily through the mitochondrial and plasma membranes. Thus their blood concentration reflects the fluxesof various mitochondrial β-oxidation steps. This process is also suspected to be protective againstthe accumulation of potentially toxic acyl-CoA derivatives in mitochondria and to preserve free CoA availability for energy metabolism [3].

In normal myocardium, the concentration of acyl-CARs is low, around 2–3 µM, because the transformation of cytosolic acyl-CoA into acyl-CAR is tightly coupled to myocardial energy demand [4]–[6]. Acyl-CARs are synthesized in the celland they can easily diffuse through the cell membrane and reach the cytosol.

Free fatty acid and acyl-CAR concentrations can increase in some pathological conditionssuch as myocardialischaemia [7], diabetes [8], [9] and genetic fatty acid disorders [10]. The arrhythmias observed in these conditions could be due to a perturbation of cardiomyocyte repolarisation by carnitine or its derivatives.

In pathological conditions such as primary systemic carnitine deficiency or ischaemia, acyl-CARs can either decrease to immeasurably low levels [2] or increase to 30 µM [11] respectively.It has been found that long chain acyl-CARs modulate the activity of certain calcium and sodium channels at a concentration of 5–10 µM [12]–[13]. However, the effects of acyl-CARs on outward currents participating in repolarization (hERG, Kv7.1/minK and KIR2.1 channels) have not been studied. Changes in the activity of these potassium channels are known to be associated with ventricular fibrillation and sudden death [14]–[15]. These arrhythmias could be either due to K^+^ channel blockade, by drugs or loss-of-function by mutations (long QT syndrome), or due to their activation by hyperkalemia, acidosis, digitalis toxicity, hyperthermia [16], or gain-of-function mutations [17].

The purpose of this study was to evaluate the effects of different concentrations of medium- and long-chain acyl-CARson these repolarising potassium channels. A physiological concentration of 3 µM acyl-CAR [5]–[6], [18], was compared to very low and high acyl-CAR concentrations, applied either intra- or extracellularly.

## Methods

### Cell Culture

HEK293 cells expressing Kv11.1 (hERG channel) were a kind gift from Drs Zhou and January (Madison, USA) [19]. HEK293(LGC Promochem, France) expressing K_v_7.1 associated with minK subunit (*KCNE1*gene) and K_IR_2.1 channel (*KCNJ2* gene, I_K1_) were made inhouse.Cells were seeded in 25 cm^2^ cell-culture flasks at 37°C and 10% CO_2_ and grown in DMEM Dulbecco’s modified Eagle’s medium supplemented with 10% (v/v) Fetal Bovine Serum (Cambrex Bio Science Verviers, Belgium).HEK-hERG medium was supplemented with 0.4 mg/ml geneticin (Gibco, Invitrogen Corporation, UK). Culture medium was changed every 2–3 days. Within 7 days, cells were at approximately 80% of confluence. The cells were then harvested using a Dulbecco Phosphate Buffered Saline without calcium and magnesium (DPBS, Cambrex Bio Science Verviers, Belgium)and trypsin (Lonza, Belgium) and seeded in a new culture flask (60000 cells/flask) for following week. Cells were also seeded in 35 mm Petri dishes to obtain a confluence of about 50%on the day of experimentation. Cells were mycoplama negative.

### Whole-cell patch–clamp Recording

Cells were placed on the stage of an inverted microscope (Nikon TE2000-U, Japan). Patch-clamp techniques have been described in detail elsewhere [20]. Currents were recorded by whole-cell voltage clamp with a Biologic RK-400 amplifier (Biologic, Grenoble, France) and an Axopatch 200 B (Axon Instrument, La Jolla, USA). Currents were filtered at 3 kHz (Axopatch) or 2 kHz (Biologic)and sampled at 5 kHz. Borosilicate glass electrodes (GC150F-15, Harvard Apparatus, UK) with tip resistances of 3.5–5 MΩ when filled with the internal solution for I_KR_ and I_K1_ channel. This solution contained (in mM): KCl 130, MgCl_2_ 1, Mg-ATP 5, EGTA 5, HEPES 10, adjusted to pH 7.2 with KOH. The internal solution for studying I_KS_ was (in mM): KCl 130, MgCl_2_ 1, NaCl 10, CaCl_2_ 1, HEPES 5, adjusted to pH 7.2 with KOH. The external physiological saline solution (PSS) contained (in mM): NaCl 137, KCl 4, MgCl_2_ 1, CaCl_2_ 1.8, D-Glucose 10, and HEPES 10, adjusted to pH 7.4 with NaOH. For HEK-I_KS_ experiments we used the perforated-patch configuration which was obtained with 0.250 mg/ml nystatin (Sigma-Aldrich, St Quentin Fallavier, France). Experiments were conducted at 23±1°C. Junction potentials were zeroed before formation of the membrane-pipette seal. Series resistance and capacitance were compensated. Current amplitudes were normalized to cell capacitance and expressed as current density (pA/pF). Cell capacitance ranged between 10 to 20pF. The cell under study was positioned at the tip of a conical microcapillary that received the outlet of six microcapillaries connected to 10 ml syringes. Superfusion was gravity-driven at 0.4 ml/min.

#### I_hERG_ protocols

Cells were held at a membrane potential of −70 mV. After control data were obtained, the superfusion was switched to the chosen acyl-CAR-containing solution, fatty-acid-containing-solution, or E4031-containing solution. Currents were elicitedby 5 s test pulses to −10 mV applied every 18 s until steady-state was obtained. Steady-state current–voltage relationships (IV) were obtained by stepping the membrane voltage for 5 s to voltages between −80 and +50 mV by 10 mV increments. Deactivating tail currents were recorded at −55 mV. The activation curves were obtained from the tail current amplitude measured at its peak value. The availability curves were obtained from the maximum current amplitude measured at a test pulse to +40 mV, applied after a two-pulse protocol that consisted of a 1 s depolarizing pulse to +40 mV followed by a second 25 ms pulse to different membrane potentials between −120 and +10 mV. At the end of each experiment, the cellunder study was perfused with E-4031 30 nM (a hERG blocker) (Alomone Labs, Jerusalem, Israel).

#### I_K1_ protocol

Cells were held at a membrane potential of −90 mV. A voltage ramp protocol of 1 s duration, from −120 to +40 mV was applied each 11 s. We measured the peak inward current, in the presence or absence of acyl-CAR. At the end of each experiment, the cell under study was perfused with BaCl_2_ 150 µM (an I_K1_ blocker) (Sigma-Aldrich, St Quentin Fallavier, France).

#### I_KS_ protocols

Cells were held at a membrane potential of −70 mV. Steady-state current–voltage relationships (IV) were obtained by stepping the membrane voltage for 3.5 s between −60 and +40 mV in 10 mV increments. Deactivating tail currents were recorded at −30 mV. The activation curves were obtained from the peak tail current amplitude. At the end of each experiment, the cell under study was perfused with Chromanol 293 B15 µM (an I_KS_ blocker) (Sigma-Aldrich, St Quentin Fallavier, France).

Command potentials, data acquisition and analysis were generated using Clampex 8.2 (Axon Instrument, Forster City, CA, USA).

Deactivating currents were best described by two exponentials using the following equation:


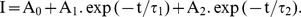


where τ_1 and_ τ_2_ are the time constants, A_1_ and A_2_ are the amplitude of the 1^st^ and 2^nd^ component of the exponential respectively and A_0_ the amplitude of the remaining current.

Half-maximal voltages (V_1/2_) of activation and availability were determined by fitting data with a Boltzmann equation:


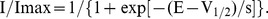


where I/Imax represents the normalized current, V_1/2_ the half-maximal voltages of activation or availability and s the slope factor. These analyses were performed using Origin 7 (Microcal Software, Northampton, MA, USA).

Acyl-CARs(except oleylcarnitine) were purchased from Drten Brink (Amsterdam, Netherland). Oleylcarnitine and chemicals were purchased from Sigma-Aldrich (St Quentin Fallavier, France). Two medium-chain acyl-CAR derivatives were used: octanoylcarnitine (C8-CAR) and decanoylcarnitine (C10-CAR) and two long-chain palmitoylcarnitine (C16-CAR) and oleylcarnitine (C18-CAR).

To simplify the terminology, we indicate the number of carbon atoms in the aliphatic chain. We used C18∶1-CARrather than C18∶0-CAR because the C18∶1 carnitine derivative is more abundant in the myocardium than C18∶0, in both physiological and pathophysiological conditions [18]. A stock solution of 30 mM Acyl-CARs, dissolved in water,was diluted to experimental concentrations between 1 and 30 µM.

### Mathematical Modelling

The model used in this study is the human endocardial ventricular cell model described by Ten Tusscher & Panvilov [21] in which we replaced the Hodgkin-Huxley equation for I_KR_ by a Markov equation.Following Fink *et al*. [22] we chose to describe I_KR_ using the following Markov chain which had 3 closed states, one open state and one inactivated state:


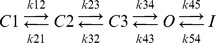


The simplex method was used to fit the experimental data using JSim software.

The reaction constants were:

k12 = 0.01507341.exp(0.001459408.V) in PSS and k12 = 0.02007341.exp(0.07865593.V) in the presence of C16-CAR.

k21 = 0.05364064.exp(−0.19822938.V) in PSS and k21 = 0.2364064.exp(−0.00011822938.V) in the presence of C16-CAR.

k23 = 0.08927204 in PSS and k23 = 0.02927204 in the presence of C16-CAR.

k32 = 15686841 in PSS and k32 = 0.01686841 in the presence of C16-CAR.

k34 = 0.01066403.exp(0.00131023.V) in PSS and k34 = 0.15066403.exp(0.0231023.V) in the presence of C16-CAR.

k43 = 0.0005743.exp(−0.0006156.V) in PSS and k43 = 0.00037433.exp(−0.04078603.V) in the presence of C16-CAR.

k45 = 0.05042237.exp(0.00854485.(V+18)) in PSS and k45 = 0.16042237.exp(0.01554485.(V+4.5)) in the presence of C16-CAR.

k54 = 0.01731608.exp(−0.04090733.(V+18)) in PSS and k54 = 0.03731608.exp(−0.05090733.(V+4.5)) in the presence of C16-CAR.

### Statistics

Data are described as mean ± standard error of the mean (n  =  number of cells).Two-way repeated measures ANOVA followed by a Student–Newmann–Keuls test were used to compare current densities. To compare the effects of acyl-CARs on the kinetics of deactivation, we performed unpaired (intracellular) and paired (extracellular) Student’s t-test. Differences were considered as statistically significant when p<0.05.

## Results

### Acylcarnitines have no Impact on I_hERG_ when Applied Intracellularly

We compared I_hERG_ in the absence or presence of intracellular free carnitine, medium-chain acyl-CARs (e.g. C8-CAR and C10-CAR), or long-chain acyl-CARs(e.g. C16-CAR and C18-CAR). Compared to the physiological concentration (3 µM), absence of acyl-CAR in the pipette had no impact on I_hERG_ (data not shown). Raising the concentration of the various acyl-CARs from 3 µM to 30 µM had no effect on the amplitude of the current (data not shown). Similarly, the kinetics of deactivation of the current were not affected by the various acyl-CARs, whatever their chain length (Table 1). We concluded that CAR and acyl-CAR had no impact on I_hERG_ when applied intracellularly.

**Table 1:** Effect of application of intra- or extracellular acyl-CARs on I_hERG_ deactivation time constants.The hERG current was fitted to a two exponential function (see Methods for details).

Treatment		Tau 1Mean ± SEM	Tau 2Mean ± SEM	n
**Control**		1663±118.5	314.4±26.5	12
**Intracellular**	3 µM C8-CAR	1378.6±75.1	263.3±12.1	8
	3 µM C10-CAR	1467.6±64.5	306.6±10.6	7
	3 µM C16-CAR	1658.4±105.8	323.5±15.8	12
	3 µM C18-CAR	1440.4±120.6	288.8±26.6	9
**Extracellular**	3 µM C8-CAR	1488.6±87.1	275.8±11.8	8
	3 µM C10-CAR	1667.9±101.1	301.5±17.8	7
	3 µM C16-CAR	1077.1±139.7**	222.2* ±21.3	7
	3 µM C18-CAR	1059.4±88.7**	212.6* ±18.9	8

The values of tau1 and tau2 are given in ms. Values were obtained when the membrane potential returned to −55 mV after stepping to −10 mV for 5s. *p<0.05, ** p<0.01.

### Long-chain Acylcarnitines Increase I_hERG_ Amplitude and Accelerate Deactivation when Applied Extracellularly

**Figure 1:**
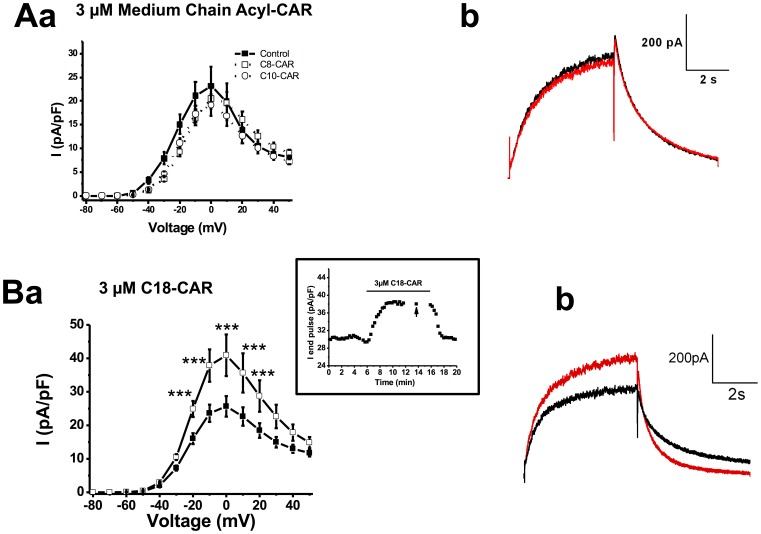
Effect of extracellularacyl-CARs on I_hERG_. I_hERG_-V relationships,in all graphs, the filled squares represent the current in the absence of acyl-CAR in the pipette (control, n = 12 cells). **Aa**, effect of 3 µM C8-CAR (empty circles, n = 8 cells) or 3 µM C10-acyl-CAR (filled circles, n = 7 cells); **Ab**, typical example of C8-CAR (red) compared with PSS (black). **Ba,** effect of 3 µM C18-CAR (filled circles, n = 9 cells) on I_hERG_-V relationship. The inset shows the effect of 3 µM C18-CAR on end-pulse hERG current elicited by a depolarisation to −10 mV from a holding voltage of −70 mV, obtained on a representative cell (the arrows indicate the current obtained at −10 mV during an IV protocol, see Methods), ***, p<0.001. **, p<0.01.**Bb**, typical example of C18-CAR (red) compared with PSS (black).

**Figure 2:**
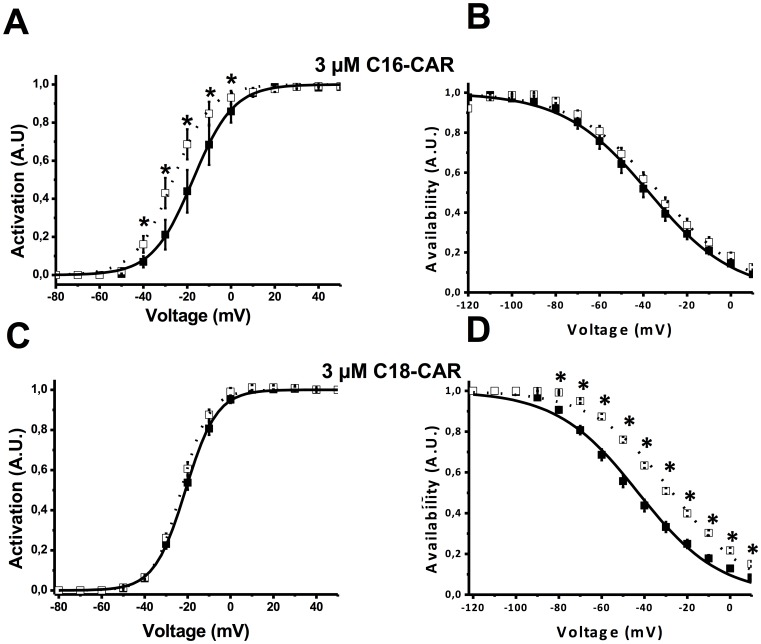
Effect of extracellular long-chain acyl-CARs on the activation and availability of the hERG current. **A**, effect of 3 µM C16-CAR on the activation curve. In the absence of C16-CAR (filled squares, n = 6 cells), the V_½_ is −17.4±0.2 mV while in the presence of 3 µM acyl-CAR (empty squares) the V_½_ is −26.5±0.5 mV. **B**, effect of 3 µM C16-CAR on availability. In the absence of C16-CAR (empty squares, n = 6 cells), the V_½_ is −35.4±1.4 mV while in the presence of 3 µM acyl-CAR (filled squares) the V_½_ is −37.4±0.5 mV. **C**, effect of 3 µM C18-CAR on activation. In the absence of C18-CAR (filled squares, n = 7 cells), the V_½_ is −21.0±0.1 mV while in the presence of 3 µM acyl-CAR (empty squares) the V_½_ is −23.0±0.2 mV. **D**, effect of 3 µM C18-CAR on availability. In the absence of C18-CAR (filled squares, n = 7 cells) the V_½_ is −42.9±1.0 mV while in the presence of 3 µM acyl-CAR (empty squares) the V_½_ is −27.0±1.0 mV. * : p<0.01.

Acyl-CARs of various chain lengths were applied extracellularly. Free CARs (data not shown) or medium-chain acyl-CARs had no impact on the amplitude of I_hERG_ (fig.1A). In contrast, 3 µM C18-CAR increased the amplitude of the end-pulse current. This effect took about 2 minutes to be complete and occurred at almost all voltages (fig. 1B). Similar results were obtained with 3 µM C16-CAR, another LCAC derivative (data not shown). External application of 3 µM C16-CAR for about 4 minutes led to a significant leftward shift of the activation curve without any effect on the availability properties (fig. 2A and 2B). Conversely, C18-CAR did not affect the activation properties while the availability curve was shifted to the right (fig. 2C and 2D). We concluded from these experiments that extracellularly-applied long-chain acyl-CAR increased I_hERG_ amplitude in a manner, dependent on their chain length.

**Figure 3:**
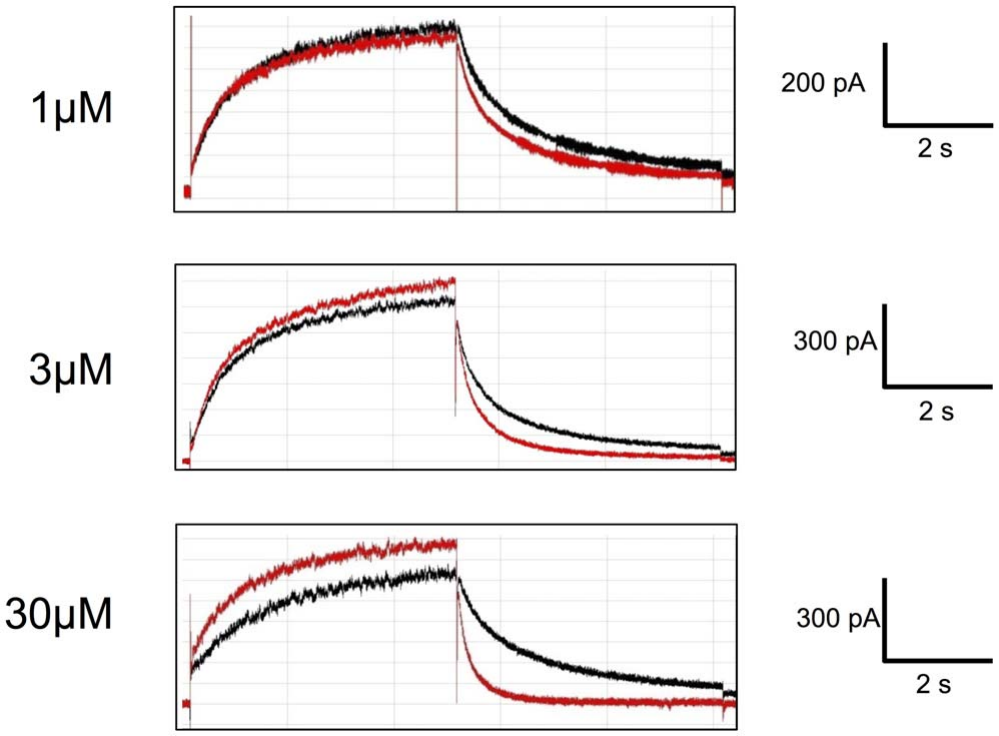
Concentration-dependent acceleration of I_hERG_ inactivation induced by C18-CAR. The records were obtained in 3 different cells. The current obtained in presence of C18-CAR is shown in red.

LCAC also affected deactivation kinetics when they are applied extracellularly, 3 µM C16-CAR and C18-CAR significantly increased deactivation kinetic (by about 30–35%), whereas medium-chain acyl-CAR had no effect on deactivation (fig. 3 and Table 1).

### The Effects of Long-chain Acylcarnitines are Dose-dependent

**Figure 4:**
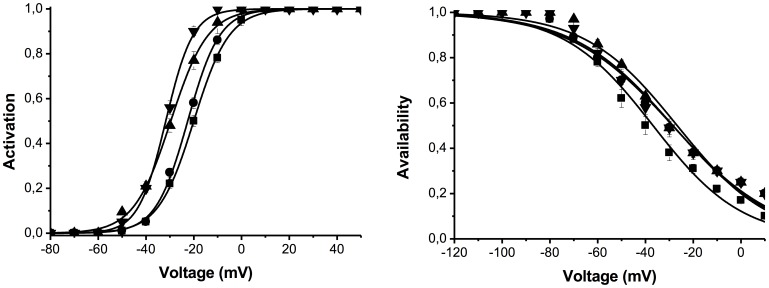
Effect of extracellular C18-CAR on hERG activation and availability. **A**. Activation curve in PSS (squares): V_½_  =  −20.3±0.1 mV (n = 10); in the presence of 1 µM C18-CAR (circles): −22.8±0.1 mV (n = 8); in the presence of 3 µM C18-CAR (diamonds): −30.0±0.3 mV (n = 8) and in the presence of 10 µM C18-CAR (triangles): −31.9±0.9 mV (n = 4). **B**. Availability curve in PSS (squares): V_½_  =  −40.9±2.3 mV (n = 8); in the presence of 1 µM C18-CAR (circles): V_½_ =  −36.2±3.1 mV (n = 8); in the presence of 3 µM C18-CAR (triangles): V_1/2_ =  −34.8±1.4 mV (n = 8) and in the presence of 10 µM C18-CAR (diamonds): V_1/2_ =  −35.6±2.1 mV.

Increasing the concentration of C18-CAR from 1 to 30 µM caused an increase in I_hERG_ amplitude (fig. 4) and deactivation kinetics (Table 2). Similar results were obtained with C16-CAR (data not shown).

**Table 2:** Effect of different concentrations of extracellular C18-CAR on I_hERG_ deactivation time constants.

Treatment	Tau1		Tau2	
	Mean ± SEM	n	Mean ± SEM	n
**PSS**	2331±160	13	469±37	13
**C18 -CAR 1** **µM**	2289±154	8	431±18	8
**C18-CAR 3** **µM**	1659±360**	4	326±72*	4
**C18-CAR 10** **µM**	772±82***	3	191±14**	3
**C18-CAR 30** **µM**	638±98***	4	176±15***	6

Values obtained when the membrane potential returned to −55 mV after stepping to −10 mV for 5 s. *p<0.05, **P<0.01, *** P<0.001.

### Accelerated Deactivation is not Due to Fatty Acid Alone

**Figure 5:**
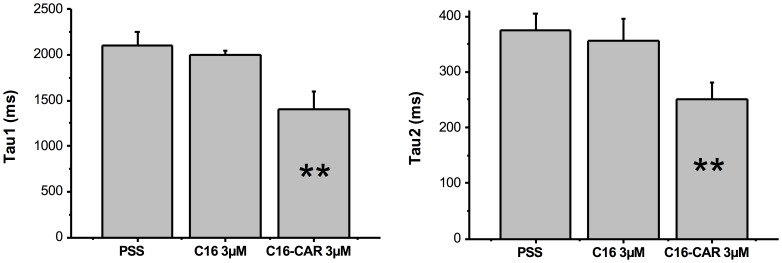
The effect of extracellular C16 or C16-CAR on deactivation kinetics of I_hERG_, when membrane potential returned to −55 mV from a 5sto −10 mV. ** p<0.01.

In order to understand the specificity of the effect of long-chain acyl-CAR on hERG channels, we investigated the effects of two long-chain free fatty acids with the same chain length. Extracellular application of 3 µM palmitoyl methyl esther (C16∶0) or oleyl methyl esther (C18∶1) caused a leftward shift of the activation curve with no change in the availability curve, reproducing increase of I_hERG_ amplitude caused by long-chain acyl-CARs (data not shown). In contrast, long-chain fatty acids did not change the deactivation kinetics (Table 3 and fig. 5).This suggests that the effect of long-chain acyl-CARs on the deactivation of I_hERG_ was specifically related to long-chain acyl-CAR derivatives.

**Table 3:** Effect of extracellular C16 free fatty acid either alone or in combination with C16-CAR on I_hERG_ deactivation time constants.

Tau1								
Voltage(mV)		PSS			C16		C16+ C16-CAR	
		*Mean ± SEM*		*n*	*Mean ± SEM*	*n*	*Mean ± SEM*	*n*
**−30**		2620.3±497.6		6	2780.7±220.7	5	2620.3±497.6	6
**−20**		2661.7±226.9		6	2131.7±148.9	5	2661.7±226.9	6
**−10**		2076.7±85.1		7	2012.8±122.1	7	2076.7±85.1	7
**0**		2070.5±133.4		7	1973.2±135.8	7	2070.5±133.4	7
**10**		2028.8±116.7		7	1927.7±113.4	7	2028.8±116.7	7
**Tau 2**								
**−30**	670.3±123.6		5		587.5±52.4	7	370.5±48.6*	7
**−20**	590.1±87.3		7		522.4±88.7	7	284.8±36.2***	7
**−10**	381.0±23.1		7		364.3±22.5	7	257.0±29.0***	7
**0**	349.3±24.2		7		337.9±25.4	7	242.0±27.3***	7
**10**	339.8±22.6		7		310.8±13.2	7	236.4±25.5***	7

Values obtained when the membrane potential returned to −55 mV after stepping to the voltage indicated in the left column in mV for 5 s. The current was fitted to a two exponential function (see Methods). The values of tau1 and tau2 are expressed in ms. *p<0.05, **P<0.01, *** P<0.001.

### Acylcarnitines do not Affect K_IR_2.1or Kv7.1/minK Channels

**Figure 6:**
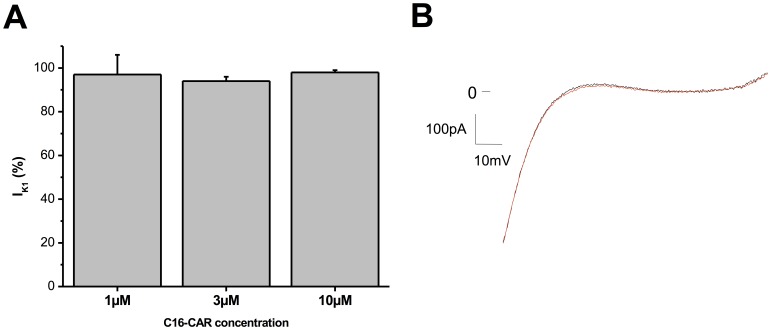
Effect of C16-CAR on I_K1_. **A**, mean current measured at −120 mV and normalized to the current in PSS (n = 7 cells). **B**, typical example of I_K1_ elicited by a ramp of voltage between −120 and +40 mV, with (red) or without (black) 10µM C16-CAR.

**Figure 7:**
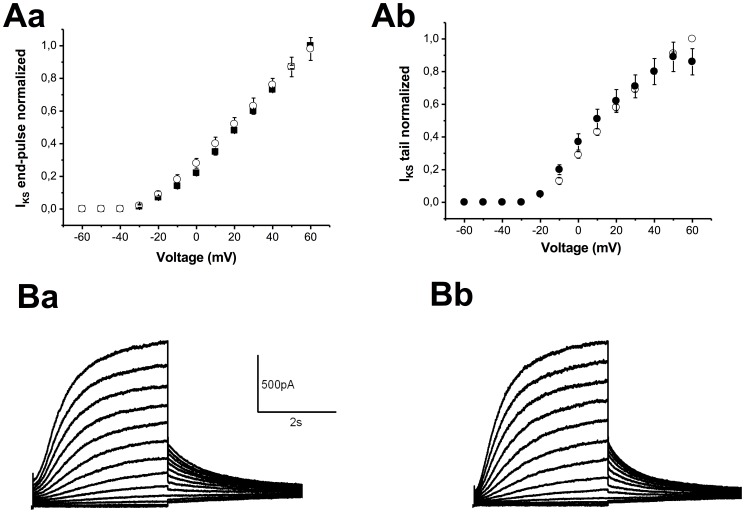
Effect of 3 µM C16-CAR on I_KS_. **A**,I_KS_-V curves of the current at the end of the pulse (**Aa**) and peak tail current (**Ab**)**. B,** Typical examples of families of currents in the absence (**Ba**) and presence of C16-CAR (**Bb**).

To evaluate a possible impact of extracellular acyl-CAR on the K_IR_2.1 channel, we compared the current in the absence or presence of long-chain acyl-CARs. At concentrations up to 10 µM, C16-CAR has no effect on I_K1_amplitude (fig. 6). Application of 3 µM C16-CAR had no effect on Kv7.1/minK channel amplitude (fig. 7) or kinetics (data not shown).

## Discussion

### Summary of Results

In this study we compare the effects of free carnitine and acyl-CARon hERG, Kv7.1/minKand K_IR_2.1 channels. Acyl-CARs with different aliphatic hydrocarbon tail lengths, were applied at different concentrations to both intra- and extracellular sides of the cell membrane. Palmitoyl-carnitine had no effect on K_IR_2.1 and Kv1.7/minK at concentrations up to 10 µM but some effects were observed on the hERG channel. These effects were specific to extracellular LCAC. Intracellular application of free carnitine or acyl-CARs had no effect on any current, irrespective of the acyl chain length. At the extracellular side of the membrane, free carnitine and medium-chain acyl-CAR up to 30 µMhad no effect. Only LCAC had some effects on hERG current.

When applied extracellularly, LCACs induced increased I_hERG_ probably due to the leftward shift of the activation for C16-CAR and rightward shift of the availability for C18-CAR. This effect began at 1 µM, a physiological concentration of LCAC, and increased dose-dependently. Since other potassium channels (K_IR_2.1 and K_V_7.1/mink) were not sensitive to LCAC, it is likely that these are direct effects on the channel protein and not due to any non-specific effects of the lipophilic compounds on the plasma membrane. These results suggest a tonic regulatory role of physiological concentrations of long-chain acyl-CAR on I_KR_.

### Effects of Long-chain Acylcarnitines on Other Ion Channels

The regulation of ion channels by acyl-CAR has been widely studied in recent years. In everycase, the comparison was between high concentrations of LCAC (up to 30 µM) and no LCAC, two situations now known to be pathological. Most of these previous studies were focused on the specific effect of C16-CAR. Various effects have been described depending on whether the acyl-CAR was applied extra-or intracellularly and also depending on the technique used to study the currents. Wu and Corr [12] found that C16-CAR applied extra- or intracellularly led to an inhibition of the whole-cell L-type calcium current in guinea-pig ventricular myocytes. Conversely, Liu and Rosenberg [23] found that 1 µM C16-CAR increased the open probability of reconstituted cardiac L-type calcium channels in membrane bilayers. I_K,ATP_ was blocked by micromolar concentrations of C16-CAR when applied to the internal face of the membrane in the inside-out configuration of the patch clamp technique [24]. Sato *et al*. [25] reported that this amphipathic molecule reduced I_K1_but only when it is applied at concentrations higher than 10 µM.Sato *et al.* reported a reversible blockade of the sodium current associated with a slowing of both activation and inactivation properties when 5 µM C16-CAR was applied extracellularly [26]. Rat cardiac ventricular I_TO_ was inhibited by C16-CAR in a dose-dependent manner when present in the pipette solution, but had no effect when superfused extracellularly [27]. In agreement with our results, these authors did not observe any effect on I_K1_ whether the amphipathic molecule was applied intra- or extracellularly at concentrations up to 10 µM.

Surprisingly, there has been no previous study of the effects of LCAC on the hERG (or I_KR_) and I_KS_ even though these channels are considered to be the target of drugs which seek to prevent ventricular fibrillation and sudden cardiac death see [15].

### Effects of Long-chain Acylcarnitines on hERG Channel

From our experiments on HEK293 cells stably expressing hERG, we can infer that LCAC regulate hERG channel activity via an extracellular site. Indeed, long-chain acyl-CARs are associated with an increase in the speed of deactivation of the channel compared to the “pathological” condition where acyl-CAR is absent. This effect is dose-dependent, at least up to 30 µM. Both, C16-CAR and C18-CAR increase the amplitude of the current but the mechanism of increased current amplitude is different. C16-CAR induced a shift of the activation curve in the hyperpolarizing direction without any effect on the availability curve. C18-CAR has no effect on the activation curve but provoked a shift in the availability curve in the depolarizing direction. Interestingly, quite similar results were obtained by Wang *et al*. [28] with lysophosphatidylcholine (LPC) having a C16∶0 or C18∶1 as acyl groups. Changing the positively charged phosphatidylcholine with phosphatidylcholineglycerol was associated with substantial modifications of the effects underlying the important role played by this charged head. These authors concluded that the effects they observed were not due to membrane incorporation of the lipids or to any intracellular signalling pathways currently known to be related to these lipids. They proposed that there must be a direct interaction of the lysophospholipid molecules with the channel protein. However, they also proposed that these lipids could modify hERG activity *via* unidentified pathways. In the present work, we found that acyl-CARs had no effect on the hERG current, at concentrations up to 30 µM. So either these lipids do not incorporate into the membrane or their incorporation has no effect on the activity of the ion channel. In contrast, LCAC can incorporate into the plasma membrane and modify membrane fluidity. However, there is no effect of the fatty acids palmitate and stearate that can also integrate the membrane. Also, LCAC does not affect K_IR_2.1 and K_V_7.1/mink currents. Thus, the electrophysiological effects of LCAC on I_hERG_ are dependent on the aliphatic hydrocarbon tail length but are unlikely to involve changes in plasma membrane properties. These results are in agreement with previously reported cases of cardiac arrhythmias associated with inherited fatty acid oxidation disorders in human. Indeed, Bonnet *et al.* [29] reported that cardiac arrhythmias and sudden death were usually observed in patients with genetic disorders of long-chain fatty acid oxidation associated with long-chain acyl-CAR accumulation. In contrast, cardiac electrical disturbances were absent in patients with medium-chain fatty acid oxidation deficiency, a condition associated with a specific increase in medium-chain acyl-CAR but normal long-chain derivatives [28].

Our observations suggest that there is a dual interaction of the acyl group and the charged head of the LPC or LCAC with the channel. Since the physiological concentration of LCAC is between 1 and 3 µM [5]–[6], [18], there must be a tonic regulation of hERG channels by long-chain acyl-CAR under physiological conditions.

### Consequences of Extracellular Accumulation or Depletion of Long-chain Acylcarnitines under Pathological Conditions

Acyl-CAR concentration can reach 10 µM in some pathological conditions such as myocardial ischaemia [7], diabetes [8], [9], or genetic fatty acid disorders [10].Conversely there is a drop in CAR and acyl-CAR cytosolic concentrations in primary systemic carnitine deficiency [2]. All these pathological situations are associated with cardiac arrhythmias and sudden cardiac death [29]–[32], [2].Accordingly, hERG regulation by LCAC could play a role in the cardiac arrhythmias genesis observed in these pathological conditions.

### Consequences for the Action Potential?

**Figure 8:**
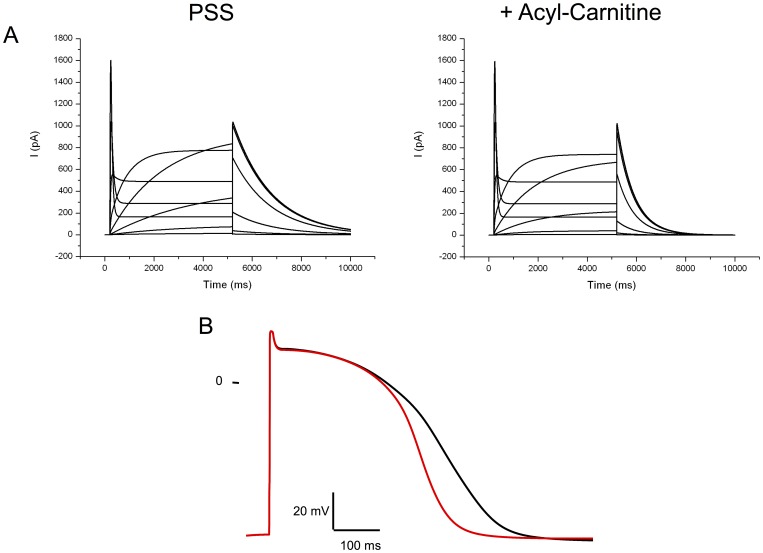
Computer simulation of the effects of LCAC on I_KR_ and the human ventricular action potential. **A**, families of currents mimicking I_KR_ with or without acyl-CARs. **B**, action potential profile in the absence of acylcarnitine(black line) or the presence of a physiological concentration of 3 µM LCAC (red line).

An increase of I_KR_, the cardiac current encoded by hERG, could lead to a reduction of the action potential duration. However, it is known that this channel inactivates and reactivates very rapidly leading to a higher current during the final repolarisation phase than during the plateau phase of the action potential. Since the deactivation of the current is accelerated, the current during the action potential must be lower. Indeed, Clancy and Rudy [33] reported a case of congenital long QT syndrome due to a mutation of hERG leading to an acceleration of I_KR_ deactivation kinetics (see also Rudy and Silva [34]). This speeding of deactivation could overcome the effect on the increased amplitude. To investigate this point, we simulated hERG current in the presence and absence of C16-CAR 3 µMusing Markov equations (see methods). Introducing such a current in the human endocardial ventricular cell model developed by TenTusscher and Panfilov [21] showed that the regulation of the hERG by C16-CAR could be responsible for a pronounced shortening of the action potential. This indicates that with the absence of carnitine, there must be an increased APD (fig. 8) while the opposite could be induced with an accumulation of LCAC, both conditions being potentially pro-arrhythmic.

### Summary

The results of this study demonstrate that long-chain acyl-CARs have regulatory properties on the hERG channel. These electrophysiological effects were not observed with medium-chain acyl-CARs. This emphasizes the relationship between long-chain fatty acid metabolism and cardiac electrical activity that contribute to cardiac arrhythmias associated with LCAC depletion or accumulation occurring in some pathologies.
